# Development and validation of a novel predictive score for sepsis risk among trauma patients

**DOI:** 10.1186/s13017-019-0231-8

**Published:** 2019-03-12

**Authors:** Hong-xiang Lu, Juan Du, Da-lin Wen, Jian-hui Sun, Min-jia Chen, An-qiang Zhang, Jian-xin Jiang

**Affiliations:** State Key Laboratory of Trauma, Burns and Combined Injury, Institute of Surgery Research, Daping Hospital, Army Military Medical University, Chongqing, 400042 China

**Keywords:** Sepsis, Trauma, Prediction, Traumatic sepsis score

## Abstract

**Background:**

Patients suffering from major trauma often experience complications such as sepsis. The early recognition of patients at high risk of sepsis after trauma is critical for precision therapy. We aimed to derive and validate a novel predictive score for sepsis risk using electronic medical record (EMR) data following trauma.

**Materials and methods:**

Clinical and laboratory variables of 684 trauma patients within 24 h after admission were collected, including 411 patients in the training cohort and 273 in the validation cohort. The least absolute shrinkage and selection operator (LASSO) technique was adopted to identify variables contributing to the early prediction of traumatic sepsis. Then, we constructed a traumatic sepsis score (TSS) using a logistic regression model based on the variables selected in the LASSO analysis. Moreover, we evaluated the discrimination and calibration of the TSS using the area under the curve (AUC) and the Hosmer-Lemeshow (H-L) goodness-of-fit test.

**Results:**

Based on the LASSO, seven variables (injury severity score, Glasgow Coma Scale, temperature, heart rate, albumin, international normalized ratio, and C-reaction protein) were selected for construction of the TSS. Our results indicated that the incidence of sepsis after trauma increased with an increasing TSS (*P*_trend_ = 7.44 × 10^−21^ for the training cohort and *P*_trend_ = 1.16 × 10^−13^ for the validation cohort). The areas under the receiver operating characteristic (ROC) curve of TSS were 0.799 (0.757–0.837) and 0.790 (0.736–0.836) for the training and validation datasets, respectively. The discriminatory power of our model was superior to that of a single variable and the sequential organ failure assessment (SOFA) score (*P* < 0.001). Moreover, the TSS was well calibrated (*P* > 0.05).

**Conclusions:**

We developed and validated a novel TSS with good discriminatory power and calibration for the prediction of sepsis risk in trauma patients based on the EMR data.

**Electronic supplementary material:**

The online version of this article (10.1186/s13017-019-0231-8) contains supplementary material, which is available to authorized users.

## Background

Trauma remains a major cause of death worldwide for individuals under 45 [1]. Although the overall mortality rate of multiple-trauma patients has declined significantly, the incidence of secondary sepsis after trauma has remained unchanged during the past decade and represents a dreaded complication [2]. Postinjury sepsis is a serious complication that typically presents a few days up to weeks after trauma, which lengthens the overall hospital stay and increases mortality [3]. Therefore, early diagnosis and treatment can improve patient prognosis and reduce mortality [4]. Bacterial culture is the standard test for diagnosing the pathogen responsible for sepsis, but prolonged culture time results in a delayed diagnosis. Moreover, blood culture tests may be negative in some patients with sepsis [5, 6]. Therefore, a new method for the early prediction of sepsis after trauma is urgently needed.

In clinical practice, clinical and laboratory variables are increasingly used for patient monitoring [7]. Automated mining of these data offers the opportunity to expedite clinical diagnoses and improve outcomes [8, 9]. Recently, Wang et al.’s study [10] derived a sepsis risk score (SRS) and severe sepsis risk score (SSRS) that accurately predicted the 10-year risk of sepsis and severe sepsis, respectively, in an adult population by taking into account the patients’ baseline individual characteristics. Lamping et al. [11] adopted a random forest technique to identify the best panel of predictors and constructed a prediction model. The model had a better predictive ability than C-reactive protein (CRP), interleukin-6 (IL-6), procalcitonin (PCT), or a combination of CRP and PCT. Furthermore, Faisal et al. [12] developed and externally validated an automated computer-aided risk score (CARS) to evaluate the risk of sepsis in emergency department patients using the patient’s baseline electronically recorded vital signs and blood test results. All of these models intend to provide an automated estimate of the risk of sepsis according to daily collected data. Because these basic information and laboratory test results are available within a few hours of admission, it is reasonable to consider that earlier recognition models could be derived for sepsis prediction based on routinely collected data.

In the current study, our objectives were to derive and validate a prediction score for the differentiation of postinjury sepsis based on routine variables that can be measured easily during clinical processes within a few hours of admission. Therefore, we followed a fully data-driven approach using all the information collected from a trauma cohort and then constructed a score for the early prediction of major sepsis in trauma patients. We hypothesized that the combination of clinical risk factors and biomarkers, compared with either alone, would provide a superior prediction model for identifying patients at high risk of sepsis after severe trauma.

## Methods

### Study populations

This prospective study was performed in the intensive care unit (ICU) of the Department of Trauma Surgery at Daping Hospital. Trauma patients were enrolled during the period from January 2005 to October 2017. Trauma patients who met the following criteria were included: (1) age between 18 and 65 years, (2) injury severity score (ISS) ≥ 16, and (3) survival time greater than 48 h in the hospital. The trauma severity of each patient was evaluated according to the ISS (The Abbreviated Injury Scale: 2005 revision) by independent clinicians. Basic features and clinical data were extracted from the electronic medical record (EMR). The diagnosis of sepsis was described as a suspected or documented infection plus an acute increase in the sequential organ failure assessment (SOFA) score by two or more points [13]. For those patients who were with multiple positive cultures, the first positive biological culture occurring during hospital period was selected. The study got approval from the Institutional Ethics Review Board of the Army Medical University, and each patient or their kin provided informed consent.

### Variables adopted for analysis and imputation

The demographic data of the enrolled patients included age, sex, drinking, smoking, and comorbidities (including obesity, hypertension, cardiac arrhythmia, and metastatic cancer). Vital signs included heart rate (HR), body temperature (BT), respiratory rate (RR), systolic blood pressure (SBP), average arterial pressure (AAP), and diastolic blood pressure (DBP). The severity of injury was determined by the AIS, ISS, and Glasgow Coma Scale (GCS). Laboratory variables included routine blood examination (white blood cell count, hemoglobin, hematocrit, platelet, neutrophil ratio, lymphocyte count, lymphocyte ratio, monocyte count, monocyte ratio, and neutrophil count), blood coagulation function, liver function tests, renal function tests, biochemical indicators, and blood gas analysis. All variables were recorded within 24 h after the patient was admitted to the hospital. If several measurements were obtained within 24 h, the worst one was adopted for the analysis. All predictors with more than 30% missing data were excluded. All missing data were imputed using a two-step approach: When a given variable was missing but there were values the day after the event, the value was used to impute the missing value. We also performed the imputation by using the mean of a variable for all other cases to replace any missing value.

### Statistical analysis

Continuous variables were reported as the mean and standard deviation (SD) and were compared between sepsis and nonsepsis patients using Student’s *t* test or Mann-Whitney *U* test. Categorical variables are presented as the number and proportion, and differences between categorical variables were compared using the chi-square test. The complete dataset was randomly separated into training and validation datasets. The training dataset contained 411 (60.0%) patients, and the remaining 273 (40.0%) patients were included in the validation dataset. To select the best predictors for the prediction model, three steps were followed to develop the risk model. First, we analyzed all potential predictors of posttraumatic sepsis risk using univariate logistic regression. Candidate predictors (*P* < 0.05) were selected through univariate analysis. To avoid introducing predictive optimism, the least absolute shrinkage and selection operator (LASSO) technique was used to select the best subset of features. Briefly, this technique shrunk the coefficient to zero by adjusting the lambda value. Just as the best subset selection, the LASSO method shrunk some coefficients of the features to zero when the lambda parameter was sufficiently large. The maximum lambda value for which the cross-validation error was within one standard error range of the minimum was selected. The LASSO regression model provides the coefficients for each feature. Moreover, a logistic regression model was constructed to convert the remaining predictors to a sepsis prediction score. After the score was derived, discrimination capability was calculated by the area under the receiver operating characteristic (ROC) curve. Model calibration was assessed by the Nagelkerke R square value and Hosmer-Lemeshow (H-L) goodness-of-fit test, with *P* > 0.05 indicating an adequate fit. The number of observed and expected sepsis morbidity case is shown. Furthermore, we compared the predictive accuracy of a single predictor and the SOFA score with our predictive score for sepsis differentiation. All statistical analyses were completed using R (version 3.3.2) and SPSS 17. *P* < 0.05 was considered statistically significant, and all statistical tests were two-sided.

## Results

### Characteristics of the study cohorts

A total of 1246 trauma patients were recorded in the dataset. According to the inclusion criteria, 562 patients were excluded; the details for exclusion are presented in Additional file 1: Figure S1. Finally, 684 trauma patients were included in our study. Of these, 411 (60.0%) were included in the training dataset, and 273 (40.0%) were included in the validation dataset. The baseline data of the patients are shown in Additional file 2: Table S1. Males comprised 331 (80.5%) and 228 (83.5%) patients in the training and validation cohorts, respectively. The patients were mostly young, with a mean age of 42.3 (12.1) and 42.6 (12.2) years. Most of the traumatic patients suffered from blunt injuries and sustained severe injuries (mean ISS 25.3 (7.7) and 25.0 (7.7)). Sepsis was usually observed at 6.4 (5.3) and 8.5 (7.4) days after the injury. The number of sepsis patients in the training and validation cohorts was 125 (30.4%) and 82 (30.0%), respectively. Gram-negative bacteria were the major microorganisms (106 (84.8%) and 70 (85.4%)). Pneumonia and primary bloodstream infection together accounted for approximately 45.6% and 57.3% of all documented infections in the training and validation groups, respectively. The SOFA score in the initial 24 h was 3.3 (2.4) and 3.6 (2.5) for the training and validation cohorts, respectively, and the average time for ICU stay was 6.1 (11.3) and 10.1 (17.8) days. The incidence of hospital death was 21 (5.1%) and 18 (6.6%) patients in the training and validation cohorts, respectively.

### Model development

**Fig. 1:**
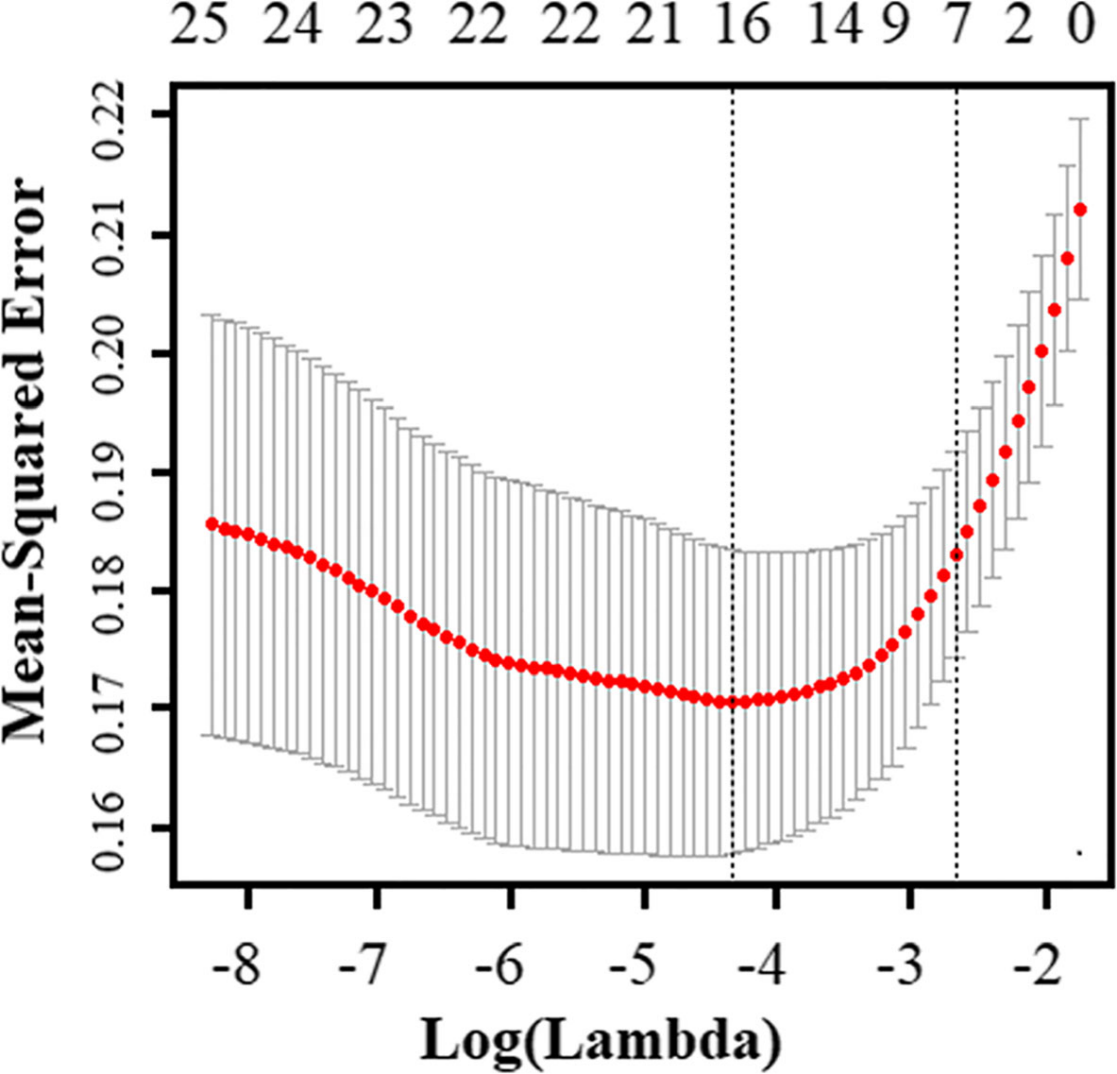
Different mean-squared error (MSE) values within the range of lambda. The MSE was calculated using the cross-validation method, and the maximum lambda parameter was selected when the cross-validation error was within one standard error range of the minimum

**Fig. 2:**
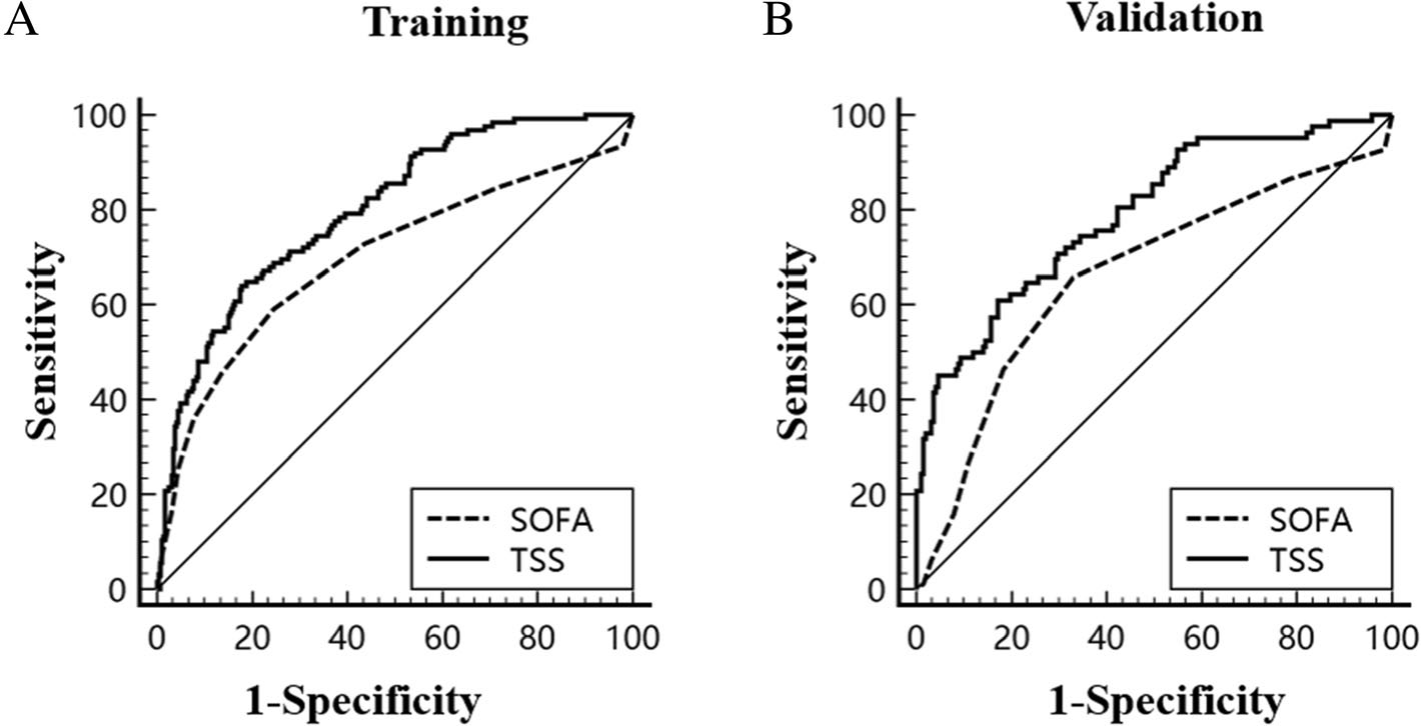
Receiver operating characteristic curve (ROC) analysis of the traumatic sepsis score (TSS) and sequential organ failure assessment (SOFA) score. **a** ROC curve analysis for the TSS and SOFA score in the training dataset (AUC = 0.799 vs. 0.698, *P* < 0.001). **b** ROC curve analysis for the TSS and SOFA score in the validation dataset (AUC = 0.790 vs. 0.662, *P* < 0.001)

**Fig. 3:**
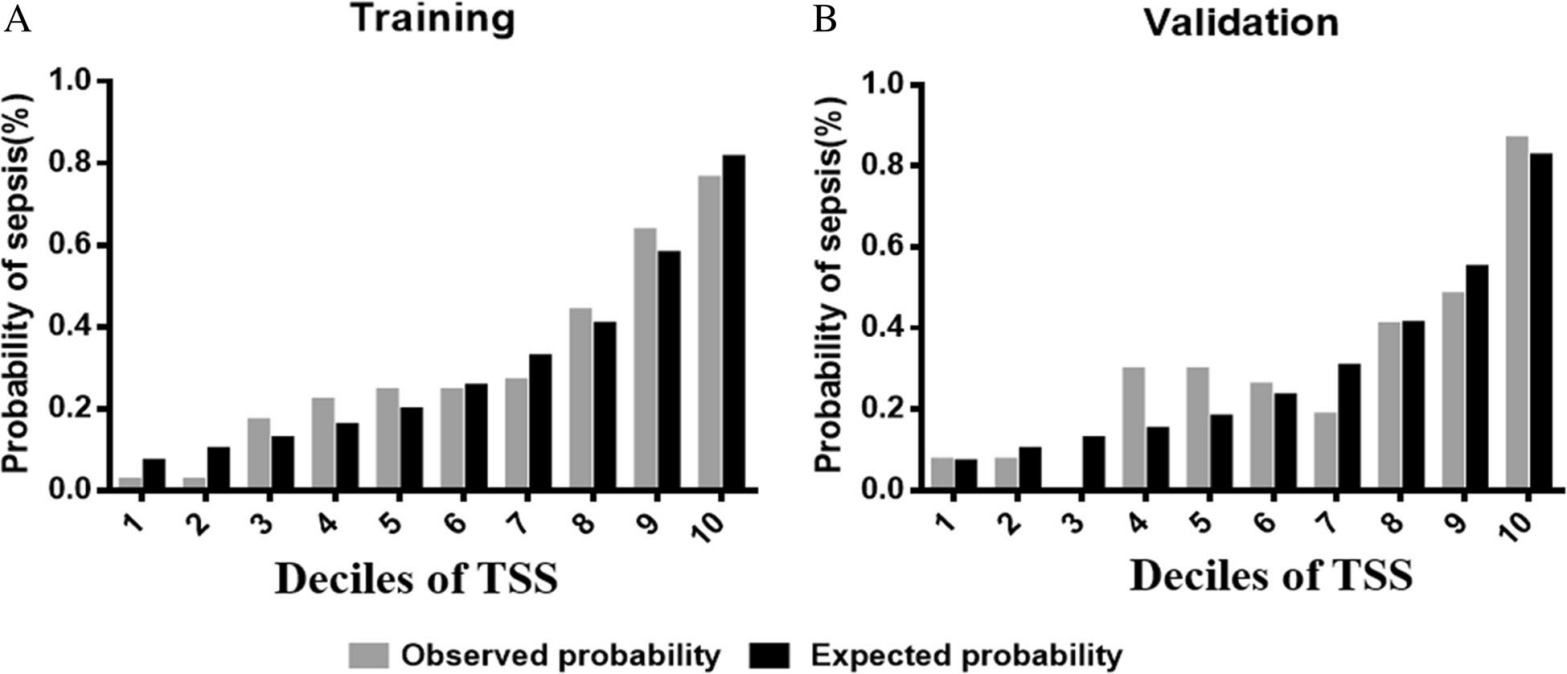
Calibration plots of the traumatic sepsis score. **a** The observed and expected probabilities of sepsis across deciles of the TSS in the training dataset. **b** The observed and expected probabilities of sepsis across deciles of the TSS in the validation dataset. (observed, gray; expected, black)

Fifty variables were considered potential predictors in the development stage of the model (Additional file 2: Table S2). We selected the variables in the training dataset using univariate analysis, and 25 predictors (*P* < 0.05) were selected for the subsequent investigation (Additional file 2: Table S2). Moreover, for generalization in clinical practice, the most suitable variables for the prediction of sepsis were selected by the LASSO technique. When LASSO was used, the different mean-squared error (MSE) values in the distinct shrinkage of parameter lambda are shown in Fig. 1. The largest lambda value was selected when the MSE cross-validation error was within one standard error of the minimum. According to the deviance, seven variables were retained in the LASSO analysis: the ISS, GCS, temperature (TP), HR, albumin (ALB), international normalized ratio (INR), and CRP (Table 1). All seven variables could be obtained within the first 24 h. The traumatic sepsis score (TSS) was calculated using the results of multivariate logistic regression results for the seven predictors (Table 1) using the following equation: TSS = [1/(1 + *e*)]-logit. We further stratified the sepsis score into four groups according to the quartile: low (score Q0–Q25), intermediate (score Q26–Q50), high (score Q51–Q75), and very high (score Q76–Q100). As shown in Table 3, 119 (28.9%) trauma patients belonged to the low-risk group, with an observational sepsis risk rate of 5.9%; 88 (21.4%) belonged to the intermediate-risk group, with a sepsis incidence of 23.9%; 103 (25.1%) belonged to the high-risk group, with an observed sepsis of 29.1%; and 101 (24.6%) belonged to the very high-risk group, with a morbidity of sepsis of 66.3% (Table 2). Therefore, with an increasing TSS, the incidence of sepsis after trauma increased in the training group (*P* for trend, 7.44 × 10^−21^). We found that the area under the curve (AUC) of the TSS was 0.799 (0.757–0.837), with 64.0% sensitivity and 82.0% specificity. Compared with individual clinical predictors, the TSS obtained a better AUC (*P* < 0.05) (Table 3). Furthermore, we compared the traumatic sepsis score with the SOFA score (AUC = 0.698 (0.638–0.758)) (Table 3), and the results showed that the TSS had a better predictive ability (*P* < 0.001) (Fig. 2a). In further support with the reliability of the TSS, the Nagelkerke R square value of TSS was 0.313 and the H-L goodness-of-fit test demonstrated no significant evidence for the TSS (*P* = 0.386). As demonstrated in the calibration plot analyses, the TSS appeared reasonably well calibrated in the training dataset (Fig. 3a).

**Table 1:** Variables for construction of TSS in training cohort (*n* = 411) (TSS = [1/(1 + *e*)]^-logit^)

Variable	LASSO coefficient	Regression coefficient (*β*)
ISS	2.77E-03	0.046
GCS	− 4.66E-03	− 0.700
TP	6.21E-02	0.536
HR	3.73E-03	0.022
INR	5.78E-02	1.012
ALB	− 7.47E-04	− 0.025
CRP	8.95E-05	0.005

*ISS* injury severity score, *GCS* Glasgow Coma Scale, *TP* temperature, *HR* heart rate, *ALB* albumin, *INR* international normalized ratio, *CRP* C-reaction protein

**Table 2:** Associations of quintile groups of TSS with incidence of sepsis

Model	Training		Validation	
	Trauma	Sepsis*	Trauma	Sepsis^#^
Sepsis score	411	125 (30.4%)	273	82 (30.0%)
≤ Q25	119	7 (5.9%)	103	12 (11.6%)
Q25–Q50	88	21 (23.9%)	52	10 (19.2%)
Q50–Q75	103	30 (29.1%)	58	19 (32.8%)
> Q75	101	67 (66.3%)	60	41 (68.3%)

*P_trend_ = 7.44E-21; ^#^*P*_trend_ = 1.16E-13

**Table 3:** Predicted probability of single variable, SOFA, and TSS

Model	AUC	Sensitivity	Specificity
Training			
ISS	0.648 (0.599–0.694)	83.20%	38.81%
GCS	0.632 (0.584–0.679)	44.80%	79.02%
TP	0.673 (0.626–0.719)	54.40%	73.78%
HR	0.731 (0.685–0.773)	62.40%	76.57%
INR	0.655 (0.607–0.701)	41.60%	83.92%
ALB	0.667 (0.619–0.712)	56.00%	73.78%
CRP	0.635 (0.586–0.682)	60.80%	60.49%
SOFA	0.698 (0.651–0.742)	59.20%	75.50%
TSS	0.799 (0.757–0.837)	64.00%	82.00%
Validation			
ISS	0.675 (0.616–0.730)	67.07%	62.30%
GCS	0.645 (0.585–0.702)	48.78%	78.53%
TP	0.657 (0.597–0.713)	41.46%	88.48%
HR	0.700 (0.641–0.753)	69.51%	64.40%
INR	0.657 (0.597–0.713)	39.02%	90.58%
ALB	0.549 (0.488–0.609)	14.63%	96.86%
CRP	0.529 (0.468–0.590)	31.71%	91.10%
SOFA	0.662 (0.603–0.718)	65.90%	67.00%
TSS	0.790 (0.736–0.836)	61.00%	83.00%

*ISS* injury severity score, *GCS* Glasgow Coma Scale, *TP* temperature, *HR* heart rate, *ALB* albumin, *INR* international normalized ratio, *CRP* C-reaction protein, *SOFA* sequential organ failure assessment, *TSS* traumatic sepsis score

### Internal validation of the TSS

To validate the TSS, we applied our sepsis score to an independent validation dataset and found that with an increasing TSS, the incidence of sepsis increased (Table 2) (*P* for trend, 1.16 × 10^−13^). Furthermore, the predictive power was estimated by area under the ROC, and the AUC was 0.790 (0.736–0.836), with 61.0% sensitivity and 83.0% specificity (Table 3). When a single predictor was included in a panel, the TSS revealed a more accurate discrimination (Table 3). In comparison with our model, the TSS also had a significantly greater discriminative ability than the SOFA score 0.662 (0.603–0.718) (*P* < 0.001) (Fig. 2b). Furthermore, no statistical evidence of the TSS was demonstrated by the H-L goodness-of-fit test (*P* = 0.082), and the Nagelkerke R square value of the TSS was 0.331 in the validation cohort. Calibration plot analyses also suggested that the TSS demonstrated reasonably good calibration in the validation dataset (Fig. 3b).

## Discussion

Dysregulation of the host inflammatory response contributes to the mortality of patients with sepsis after trauma [14–16]. Despite innovations in therapy, the mortality rate remains very high due to sepsis and subsequent complications. Therefore, identifying trauma patients at high risk of sepsis is of utmost importance to provide appropriate and timely interventions to reduce mortality [17]. In contrast to current acute care paradigms, understanding an individual’s susceptibility to sepsis could provide important opportunities to reduce the long-term risk and the public health burden [2, 18]. In the current study, we found that it was feasible to develop a novel predictive scoring system for traumatic sepsis based on routinely available data. In our study, the LASSO technique was implemented to select the best factors for the prediction score. Then, we derived the score from the training dataset using logistic regression and externally validated it in other datasets. We demonstrated that the discrimination and calibration of the score are reasonably good. Our results also suggest that the TSS has better predictive power than any single predictor or the SOFA score.

In the current study, seven predictors were included. The advantage of our traumatic sepsis score is that it incorporates seven components, each of which is independently strongly associated with traumatic sepsis. The ISS was used to reflect the severity of the injury and indicates tissue damage. The ISS has been validated as an independent predictor of the risk for traumatic sepsis, and trauma patients with higher ISS scores have been shown to have a greater incidence of sepsis [19]. Initially used as an evaluation tool for patients with head injury, the GCS has become an important component of severity systems after injury. Patients with severe neurological injury have a higher occurrence of sepsis, severe sepsis, and septic shock [1, 20]. As parts of the SIRS standard, abnormal BT and HR are usually considered markers of infection, despite a lack of documentation of their accuracy. Abnormal BT curves are predictive of a diagnosis of sepsis in critically ill patients. Meanwhile, previous studies have suggested that abnormal HR characteristics provide information beyond that of laboratory tests for the diagnosis of culture-positive neonatal sepsis [21]. Because of the pathophysiological processes that occur after major injury, it is not surprising that three physiologic parameters (ALB, INR, and CRP) are incorporated into our score. Malnutrition increases the risk of surgical-site infection, pneumonia, and urinary tract infection, and serum ALB is the most well-established serum marker of malnutrition. In comparison with patients who have normal serum ALB, patients with hypoalbuminemia have a higher rate of sepsis [22]. As coagulopathy on presentation is a stronger predictor of sepsis [23], elevated INR measurements may allow for the early identification of patients who are at risk for sepsis. Furthermore, major trauma patients usually present bleeding and require blood transfusion, which can affect serum ALB and INR [24]. Thus, it is reasonable to assume that these two indicators can be used to predict the risk of sepsis after injury. CRP is an acute phase protein that is elevated in trauma patients, and numerous studies have suggested that CRP can be utilized to predict the development of sepsis [17, 25, 26].

From the above findings, we conclude that the seven clinical variables and biomarkers included in our prediction model are independent risk factors for sepsis in trauma patients. Although any single variable could be applied for the early prediction of the risk of sepsis in these patients, the discriminatory ability of a single variable is limited. When these variables are integrated into a panel, the predictive ability greatly improves. The identification of individuals who are at high risk of sepsis aids in clinical decision-making regarding targeted treatment strategies. At present, the occurrence of sepsis in trauma patients can be evaluated using the SOFA score, but it requires the parameters of multiple variables, such as PaO2, platelet count, creatinine, and bilirubin levels [27]. In our current study, TSS incorporates objective and commonly measured variables, including the ISS, GCS, TP, HR, ALB, INR, and CRP. The TSS is designed to accurately predict incident sepsis after trauma, and it can enhance resource utilization through the targeted treatment of patients with the highest risk of sepsis. The clinical application of the TSS can improve outpatient care or early hospital care for patients with infections and promote the earlier use of antibiotic therapy.

The present study has several obvious strengths. Multivariate regression models are traditionally performed for early predictions in clinical research due to their simplicity. In the current study, the LASSO technique was more suitable for variable selection because of the availability of complex medical data. Moreover, the seven selected predictors are available within several hours of admission and could be combined into a panel quickly. The study also had some disadvantages. First, it was performed at a single medical center. Although the TSS was verified in the validation cohort, the importance of external validation with sufficient data cannot be ignored. Second, the sensitivity and specificity of the TSS are only moderately good, with an AUC of 0.799. Some variables related to sepsis are not incorporated into the predictive score, probably due to insufficient power or missing values. Additionally, in our study, only variables during the first 24 h after admission were included. Other risk factors for sepsis, such as antibiotic use, blood transfusion, and tracheal cannula, were not incorporated into our score [15]. When more risk variables are included in the TSS, then its predictive ability will improve. Finally, our prediction model is static. It is more reasonable to mine the association between time series variables and the risk of sepsis.

## Conclusions

Our study identified seven clinical parameters and biomarkers as predictors for traumatic sepsis and then derived a combination score. Our TSS provides good discrimination and calibration for predicting the occurrence of sepsis in trauma patients. Once validated externally, our model may be applied to identify patients at risk of sepsis after injury.

## Additional files

Additional file 1: **Figure S1.** Flowchart of patient selection. A total of 684 trauma patients were enrolled in the current study, including 411 in the training cohort and 273 in the validation cohort. (TIF 156 kb)

Additional file 2: **Table S1.** Baseline characteristics of trauma patients in training and validation cohorts*. Table S2. Baseline characteristics and clinical tests included in the training cohorts. (DOCX 23 kb)

## Abbreviations

ALB: Albumin
AUC: Area under the curve
CRP: C-reaction protein
GCS: Glasgow Coma Scale
HR: Heart rate
IL-6: Interleukin-6
INR: International normalized ratio
ISS: Injury severity score
LASSO: Least absolute shrinkage and selection operator
PCT: Procalcitonin
ROC: Receiver operating characteristic curve
SIRS: Systemic inflammatory response syndrome
SOFA: Sequential organ failure assessment
TP: Temperature
TSS: Traumatic sepsis score
