# Supplementary figures and images for: Development and validation of a novel predictive score for sepsis risk among trauma patients
Source: World J Emerg Surg. 2019 Mar 12;14:11. doi: 10.1186/s13017-019-0231-8 (PMC6419404; doi:10.1186/s13017-019-0231-8)

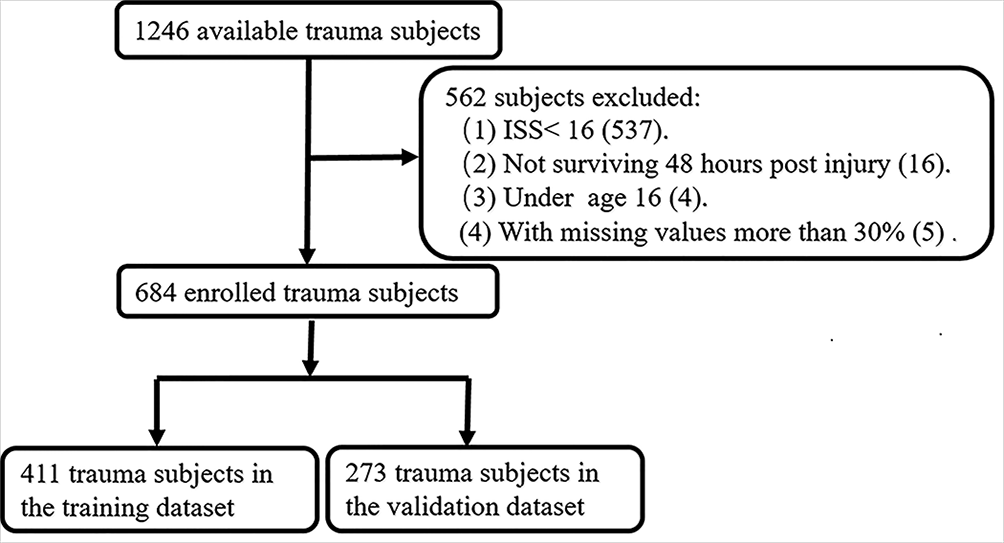

Supplement: Supplementary file 1 — Figure S1. Flowchart of patient selection. A total of 684 trauma patients were enrolled in the current study, including 411 in the training cohort and 273 in the validation cohort. (TIF 156 kb) [file 13017_2019_231_MOESM1_ESM.tif]
