# Supplementary material for: Development and validation of a novel predictive score for sepsis risk among trauma patients
Source: World J Emerg Surg. 2019 Mar 12;14:11. doi: 10.1186/s13017-019-0231-8 (PMC6419404; doi:10.1186/s13017-019-0231-8)
Supplement: Supplementary file 2 — Table S1. Baseline characteristics of trauma patients in training and validation cohorts*. Table S2. Baseline characteristics and clinical tests included in the training cohorts. (DOCX 23 kb) [file 13017_2019_231_MOESM2_ESM.docx]

**Table S1.** Baseline characteristics of trauma patients in training and validation cohorts*.

| **Variables** | **Training (n = 411)** | **Validation (n = 273)** | **P value** |
| --- | --- | --- | --- |
| Female/Male | 80/331 (19.5%/80.5%) | 45/228 (16.5%/83.5%) | 0.323 |
| Age (years) | 42.3( 12.1) | 42.6 ( 12.2) | 0.681 |
| AIS Head/neck | 1.9 (1.7) | 2.3( 1.7) | <0.001 |
| AIS Face | 0.4(0.8) | 0.4 ( 0.7) | 0.284 |
| AIS Thorax | 2.8 (1.3) | 2.6 (1.3) | 0.059 |
| AIS Abdomen | 1.4 (1.6) | 1.2 (1.4) | 0.148 |
| AIS Upper/lower extremity | 2.1( 1.5) | 1.8(1.6) | 0.015 |
| ISS | 25.3(7.7) | 25.0(7.7) | 0.320 |
| GCS initial | 13.4(2.6) | 12.4 ( 3.8) | 0.001 |
| GCS max | 13.9(2.8) | 13.2(3.3) | 0.017 |
| Admission time after injury (hours) | 33.7 (76.6) | 45.8( 109.0) | 0.112 |
| Initial SBP | 120.9(17.8) | 120.1(21.9) | 0.594 |
| Mechanical ventilation (days) | 5.2 (11.2) | 11.1 (11.2) | <0.001 |
| Total pRBC transfusion (ml) | 88.6( 346.6) | 68.5(309.5) | 0.438 |
| RTS | 11.5 (1.02) | 11.2 (1.2) | 0.001 |
| APACHE II initial | 8.4(7.2) | 10.2(6.4) | 0.001 |
| APACHE II max | 11.5(7.8) | 12.9(7.3) | 0.17 |
| SOFA initial | 3.3(2.4) | 3.6(2.5) | 0.367 |
| SOFA max | 4.2(2.9) | 4.7(3.0) | <0.001 |
| Sepsis | 125 (30.4%) | 82 (30.0%) | 0.916 |
| Pathogens n (%) |  |  |  |
| Gram-negative | 106 (84.8%) | 70 (85.4%) |  |
| Gram-positive | 13 (10.4%) | 2 (2.4%) | <0.001 |
| Others | 6 (4.8%) | 10 (12.2%) |  |
| Source of infection n (%) |  |  |  |
| Blood | 31 (24.8%) | 28 (34.1%) |  |
| Sputum | 26 (20.8%) | 19 (23.2%) |  |
| Urine | 32 (25.6%) | 15 (18.3%) | 0.007 |
| Secretions | 27 (21.6%) | 7 (8.5%) |  |
| Others | 8 (6.4%) | 13 (15.9%) |  |
| Time trauma-sepsis onset | 6.4(5.3) | 8.5(7.4) | 0.02 |
| ICU days | 6.1(11.3) | 10.1(17.8) | <0.001 |
| Mortality | 21 (5.1%) | 18 (6.6%) | 0.412 |

*AIS: Abbreviated injury scale; ISS: Injury severity score; GCS: Glasgow Coma Scale; RTS: Revised trauma score; APACHE II: Acute Physiology and Chronic Health Evaluation; SOFA: Sequential Organ Failure Assessment; ICU: Intensive Care Unit.

**Table S2. Baseline characteristics and clinical tests included in the training cohorts.**

| **Variable** | **Sepsis (n=125)** | **Non-sepsis (n=286)** | **P value** |
| --- | --- | --- | --- |
| Female/Male | 25/100 (20.0%/80.0%) | 55/231 (19.2%/80.8%) | 0.856 |
| Age (years) | 43.2(12.4) | 42.9(12.0) | 0.834 |
| Time after injury (hour) | 29.9(38.9) | 35.3(88.1) | 0.518 |
| Initial SBP | 120.6(19.9) | 121.0(16.9) | 0.841 |
| Any PRBC | 33 (26.4%) | 60 (21.0%) | 0.228 |
| ISS | 27.9 (8.5) | 24.2(7.0) | <0.001 |
| GCS initial | 12.1(4.2) | 14.0(2.2) | <0.001 |
| Smoking | 40 (32.0%) | 94 (32.9%) | 0.863 |
| Drinking | 17 (13.6%) | 45 (15.7%) | 0.578 |
| Basic diseases | 10 (8.0%) | 23 (8.0%) | 0.989 |
| C-reactive protein (CRP) | 80.0(55.7) | 55.3(49.0) | <0.001 |
| **Vital sign** |  |  |  |
| Temperature (TP) | 37.6(0.8) | 37.1(0.5) | <0.001 |
| Heart rate (HR) | 111.2(23.8) | 93.3(18.1) | <0.001 |
| Respiratory rate (RR) | 22.0(4.9) | 20.8(2.8) | 0.003 |
| Systolic blood pressure (SBP) | 121.7(20.8) | 123.4(17.6) | 0.397 |
| Diastolic blood pressure (DBP) | 72.7(15.9) | 75.4(12.6) | 0.071 |
| Average arterial pressure (AAP) | 89.0(16.5) | 91.4(13.2) | 0.128 |
| **Blood routine examination** |  |  |  |
| White blood cell count (WBC) | 12.4(5.8) | 11.8(4.9) | 0.233 |
| Hemoglobin (HGB) | 98.6(25.3) | 109.3(22.9) | <0.001 |
| Hematocrit (HCT) | 29.0 (7.4) | 32.3(6.6) | <0.001 |
| Platelet (PLT) | 122.8(71.7) | 142.3(55.8) | 0.003 |
| Neutrophil ratio (N%) | 86.5(5.6) | 84.7 (7.3) | 0.019 |
| Lymphocyte count (LYMPH) | 0.8(0.5) | 0.9(0.4) | 0.045 |
| Lymphocyte ratio (LYMPH%) | 8.4(5.4) | 9.3(5.1) | 0.106 |
| Monocyte count (MONO) | 0.7(0.4) | 0.7(0.9) | 0.463 |
| Monocyte ratio (MONO%) | 5.8(2.6） | 5.9(2.3) | 0.627 |
| Neutrophil count (NEUT) | 10.7(5.3) | 10.0(4.2) | 0.146 |
| **Coagulation function** |  |  |  |
| International normalized ratio (INR) | 1.1(0.3) | 1.0(0.2) | <0.001 |
| Activated partial thromboplastin time (APTT) | 37.9(26.3) | 30.3(10.4) | 0.001 |
| Thrombin time (TT) | 17.8(10.1) | 16.7 (7.8) | 0.21 |
| Prothrombin time (PT) | 13.8(5.7) | 11.8(2.3) | <0.001 |
| D-Dimer (DD) | 6586.3(7930.8) | 5201.1(6590.0) | 0.075 |
| **Liver function** |  |  |  |
| Albumin (ALB) | 27. 8(7.6) | 31.7(6.3) | <0.001 |
| Total bilirubin (BIL) | 21.4(14.8) | 19.4(8.7) | 0.092 |
| Aspartic acid aminotransferase (AST) | 279.7(610.4) | 181.3(1227.6) | 0.434 |
| Alanine aminotransferase (ALT) | 154.3(341.6) | 122.6(763.5) | 0.663 |
| **Renal function** |  |  |  |
| Urea | 7.2 (4.1) | 6.0(4.4) | 0.027 |
| Creatinine (Cr) | 98.4(84.5) | 78.7(88.9) | 0.089 |
| **Biochemical indicators** |  |  |  |
| Potassium (K) | 3.9(0.6) | 3.8(0.5) | 0.024 |
| Sodium (Na) | 138.9(5.4) | 137.8(3.7) | 0.018 |
| Chlorine (Cl) | 106.3(5.7) | 104.7(4.6) | 0.004 |
| Calcium (Ca) | 2.0(0.2) | 2.1(0.2) | 0.008 |
| **Blood gas** |  |  |  |
| PH | 7.4(0.1) | 7.4(0.1) | 0.069 |
| Partial pressure CO2 (CO2) | 35.9(6.3) | 36.5(5.1) | 0.354 |
| Partial pressure O2 (PO2) | 122.7(79.1) | 118.6(60.0) | 0.569 |
| Bicarbonate (HCO3) | 22.8(3.7) | 24.0(3.0) | 0.001 |
| Base excess (BE) | 1.6(4.3) | 0.2(3.3) | <0.001 |
| Oxygen saturation (SatO2) | 94.7(10.5) | 96.4(3.8) | 0.032 |
| Oxygen index (PaO2/FiO2) | 3.4(2.1) | 4.0(1.9) | 0.002 |
| Lactic acid (LAC) | 2.4(1.9) | 1.8(1.6) | 0.003 |
